# Parents’ attitudes toward childhood COVID-19 immunization in Croatia: a multicenter cross-sectional study

**DOI:** 10.3325/cmj.2023.64.52

**Published:** 2023-02

**Authors:** Mirta Šašić, Kristian Bodulić, Iva Hojsak, Mario Mašić, Ivana Trivić, Joško Markić, Marijan Batinić, Ines Bartulović, Anja Šurina, Nina Krajcar, Goran Tešović

**Affiliations:** 1 Pediatric Infectious Diseases Department, Dr. Fran Mihaljević University Hospital for Infectious Diseases, Zagreb, Croatia; 2 Research Department, Dr. Fran Mihaljević University Hospital for Infectious Diseases, Zagreb, Croatia; 3 Referral Center for Pediatric Gastroenterology and Nutrition, Children’s Hospital Zagreb, Zagreb, Croatia; 4 Department of Pediatrics, University Hospital Center Split, University of Split, School of Medicine, Split, Croatia; 5 Department of Pediatrics, University Hospital Center Split, Split, Croatia; 6 Department of Pediatrics, University Hospital Centre Osijek, Osijek, Croatia; 7 University of Zagreb, School of Medicine, Zagreb, Croatia; The first two authors contributed equally.

## Abstract

**Aim:**

To assess parents’ attitudes toward childhood COVID-19 immunization in Croatia.

**Methods:**

In this multicenter cross-sectional study, we collected data from four tertiary care facilities in Zagreb, Split, and Osijek between December 2021 and February 2022. During the visit to the Pediatric Emergency Departments, parents were asked to fill out a highly-structured questionnaire about their attitudes toward COVID-19 immunization in children.

**Results:**

The sample consisted of 872 respondents. A total of 46.3% of respondents were hesitant about vaccinating their child against COVID-19, 35.2% definitely did not intend to vaccinate their child, and 18.5% definitely intended to vaccinate their child. Parents who were themselves vaccinated against COVID-19 were more likely than unvaccinated parents (29.2% and 3.2%, *P* < 0.001) to vaccinate their children. Parents agreeing with the epidemiological guidelines were more inclined to vaccinate their children, as were parents of older children and parents of children vaccinated according to the national program schedule. Child comorbidities and respondents’ history of COVID-19 were not associated with childhood vaccination intention. Ordinal logistic regression revealed that the most important predictors for a positive parents’ attitude toward vaccinating their child were parents’ vaccination status and regular vaccination of their child according to the national immunization program schedule.

**Conclusion:**

Our results demonstrate Croatian parents’ mostly hesitant and negative attitudes toward childhood COVID-19 immunization. Future vaccination campaigns should target unvaccinated parents, parents with younger children, and parents of children with chronic diseases.

Most of the children who contract SARS CoV-2 infection (1) are asymptomatic or present with mild to moderate symptoms (2). Clinical manifestations of COVID-19 in children may be less severe than in adult patients. The factors associated with severe outcomes in children are younger age, underlying pulmonary pathology, and immunocompromising conditions (3,4). Other risk factors for severe COVID-19 and hospitalization include obesity, type-1 diabetes, prematurity, and chronic diseases such as congenital heart diseases (5). Although COVID-19 is generally considered a mild disease in children, a small proportion of patients develop severe disease, requiring intensive care unit (ICU) admission and prolonged ventilation. Fatal outcomes are overall rare (6).

The new coronavirus pandemic was met with rapid development of vaccines. Mass vaccination campaigns against COVID-19 are ongoing worldwide. Vaccines are effective tools for decreasing the number of severely ill and hospitalized patients. Among numerous advantages, the most important rationale for vaccinating adolescents and younger children is their protection against COVID-19 (7). Notably, SARS-CoV-2 infection in children has similar characteristics to influenza, for which effective vaccines are available and strongly recommended worldwide. Groups of pre-adolescents and adolescents with COVID-19 and influenza had similar hospitalization rate, intensive care unit admission rate, and need for mechanical ventilation (8). Another potential advantage of childhood COVID-19 immunization is reducing SARS-CoV-2 transmission in the community. Similar trends have already been demonstrated for the influenza vaccine and brought enormous medical, social, and economic advantages (9). The reduction in SARS-CoV-2 transmission rates has become exceptionally important when considering the waning post-infection and post-vaccination immunity and the high transmission rates of the Omicron variant (10,11). However, the role of children in spreading SARS-CoV-2 is still unclear, with several studies demonstrating lower SARS-CoV-2 household transition rates in children (12,13). Furthermore, in many countries the proportion of children among the confirmed COVID-19 cases is considered low (14). However, some studies indicate that these epidemiological patterns could be explained by a reduced testing rate in children due to mild symptoms and lower susceptibility to infection compared with adults (15). The role of children in SARS-CoV-2 transmission is highly dependent on the circulating viral variants (13,16). Corresponding to trends found in adults, the Omicron variant exhibits significantly higher transmission rates in children (13,16). Considering the stated factors, the role of children in SARS-CoV-2 transmission remains unknown.

Parents’ attitudes toward childhood vaccination and vaccination coverage among children have been the topic of interest in various studies (17,18). Many authors investigated the sociodemographics and sociocultural determinants of vaccination refusal and hesitancy in different countries (19,20). Similar studies have also been conducted in Croatia, demonstrating an overall positive opinion on childhood vaccination according to the national immunization program schedule, with parents’ age, gender, and education being not significantly associated with attitude toward childhood vaccination (21,22). Considering a recent rise in the vaccination refusal rate in Croatia (23), there is a valid presumption of even higher refusal and hesitancy rates given a novel vaccine introduction. At the time of writing, there are no studies dealing with parents’ attitudes toward childhood COVID-19 vaccination in Croatia. In light of the current COVID-19 pandemic, this study aimed to estimate the rate and the predictors of parents’ acceptance of childhood immunization against COVID-19 in Croatia.

## Respondents and methods

### Respondents, questionnaire, and data collection

A multicenter, cross-sectional study was performed in four university hospitals in three Croatian regional centers (Zagreb, Split, and Osijek) from December 2021 to February 2022. For data collection, we developed a highly structured questionnaire on parents’ attitudes toward COVID-19 immunization in children. The questionnaire was adapted for self-completion and was given to all parents who visited the Pediatric Emergency Units during the study period. Participation in the study was voluntary, and the respondents were informed that the anonymity of their responses was protected. The number of respondents was not pre-determined, but depended on the study duration. Questions regarding the participants’ sociodemographic characteristics were adopted from Yang et al (24). Apart from questions related to parents’ sociodemographic status, various other questions were included to better examine the factors possibly related to parents’ attitudes toward vaccinating their child against COVID-19. The study was approved by the Dr Fran Mihaljević University Hospital for Infectious Diseases Ethics Committee before data collection.

### Outcome variable

The outcome variable was the answer to the question: “How likely are you to vaccinate your child against COVID-19?”. The possible answers were 1) “I will definitely vaccinate my child against COVID-19“, 2) “I’m hesitant about vaccinating my child against COVID-19“, 3) “I’m less likely to vaccinate my child against COVID-19“, and 4) “I will definitely not vaccinate my child against COVID-19”. The outcome variable was later recategorized into a three-level factor, with the second and third answer combined into the hesitant group.

### Independent variables

The surveying center was a three-level categorical variable (1 = Zagreb, 2 = Split, 3 = Osijek). Respondents’ age was a numerical variable recategorized into a three-level categorical variable (1 = 18-30 years, 2 = 31-45 years, and 3 = more than 45 years). Sex and place of residence were two-level indicators (sex: 1 = male, 2 = female; place of residence: 1 = urban, 2 = rural). The number of household members was recategorized into a two-level categorical variable (1 = 2-4 members and 2 = more than 4 members). Education level was measured on a six-point scale, and was later recategorized into a two-level factor (1 = secondary education or lower and 2 = tertiary education or higher). Likewise, monthly household income was measured on a seven-point scale and was later recategorized into a two-level factor (1 = less than €2000 and 2 = more than €2000). COVID-19 infection history of household members, respondent’s COVID-19 vaccination status, and post-vaccination side-effect status were two-level indicators (1 = yes 2 = no), while post-vaccination side-effect type was a three-level variable (1 = local, 2 = systemic, 3 = local and systemic). Adherence to the epidemiological measures was measured on a five-point scale, ranging from 1 = completely not adhering to the epidemiological measures to 5 = completely adhering to the epidemiological measures. This variable was later reclassified into a two-level factor due to a skewed distribution (1 = not completely adhering to the epidemiological measures and 2 = completely adhering to the epidemiological measures). Similarly, agreement with the epidemiological measures was measured on a five-point scale, ranging from 1 = completely disagreeing with the epidemiological measures and 5 = completely agreeing with the epidemiological measures. This variable was also reclassified as a two-level factor due to a skewed distribution (1 = not completely or partially agreeing with the epidemiological measures and 2 = partially or completely agreeing with the epidemiological measures). Child age was a numerical variable later transformed into a three-factor categorical variable (1 = 0-5 years, 2 = 6-11 years, and 3 = 12-18 years). Child comorbidities, history of COVID-19, and vaccination according to the national immunization program schedule were reclassified into two-level indicators (1 = yes, 2 = no).

### Statistical analysis

The respondents were weighted by raking to account for the difference between the sample and the adult population in Croatia. The variables used in raking included sex, education level, and the child’s age. The respondents were also weighted to reflect the population size of the counties covering the three sampling centers analyzed in this study. The maximum weight was set to 5. Data on the population’s age structure and county population were retrieved from the first results of the 2021 census (28). Data on the population’s educational structure were retrieved from the 2011 census (29). Respondents with missing values for variables used in raking or for the outcome variable were excluded from the analysis (N = 102). Childhood vaccination intention was compared among different groups of interest using the χ^2^ test with Rao-Scott correction. *P* values were adjusted for multiple testing with the Bonferroni method. Ordinal logistic regression was used to analyze the predictors of vaccination intention in a multivariate setting. All predictors significantly associated with vaccination intention in the bivariate analysis were included in the model. Respondents who did not intend to vaccinate their child were set as the reference group. The coefficients’ confidence intervals were estimated by likelihood function profiling. The proportional odds assumption was evaluated by the Brant-Wald test. *P* values lower than 0.05 were considered significant, and all statistical tests were two-tailed. Statistical analysis was performed in R (version 4.1.0) (25) with anesrake (version 0.8.0.) (26) and survey packages (version 4.1.1.) (27).

## Results

### Sample characteristics

A total of 974 completed questionnaires were collected. Hundred and two questionnaires were excluded due to missing data, so the final sample consisted of 872 respondents. The assigned sampling weights ranged from 0.23 to 5.00 (mean = 1.00, SD = 1.29). After weighting, 51.8% of respondents were female (mean age of 39.9 years; SD = 7.8 years). Considering education, 83.6% of respondents had completed secondary education or less, while 16.4% had completed tertiary education. Additionally, 61.0% of respondents were surveyed in Zagreb, 24.2% in Split, and 14.8% in Osijek. The mean age of the respondents’ children was 9.1 years (SD = 5.5 years). In terms of COVID-19 vaccination status, 59.2% of respondents were vaccinated. Furthermore, 61.0% of respondents reported that a member of their household had contracted COVID-19 before surveying. Additionally, 28.5% of respondents reported that their child had recovered from COVID-19 before surveying. Finally, 91.5% of the respondents stated that their child had been vaccinated regularly according to the national immunization program schedule.

### Bivariate analysis of childhood vaccination intention

Overall, 46.3% of the respondents were hesitant to vaccinate their child against COVID-19 (95% CI, 41.9%-50.7%), 35.2% reported that they would definitely not vaccinate their child (95% CI, 31.9%-39.3%), and 18.5% reported that they would definitely vaccinate their child (95% CI, 15.0%-22.0%). We carried out several bivariate analyses to assess the relationship between predictors of interest and vaccination intention (Table 1). Respondents’ age was positively associated with vaccination intention (*P* = 0.009), with respondents older than 45 years being more likely to vaccinate their child (31.2%) than those in the 18-30 years group (17.5%) and 31-45 years group (14.5%). Respondents vaccinated against COVID-19 were more likely to vaccinate their child than respondents who were not vaccinated against COVID-19 (29.2% and 3.2%, *P* < 0.001). This finding was similar in all age groups (18-30 years: 31.1% and 2.5%, *P* < 0.001; 30-45 years: 25.7% and 2.8%, *P* < 0.001; >45 years: 34.5% and 5.7%, *P* < 0.001). Moreover, child vaccination intention was not significantly associated with age groups when stratified by respondents’ vaccination status (*P* > 0.05). In addition, the adherence to epidemiological measures was positively correlated with vaccination acceptance, where respondents who fully complied with the epidemiological measures were more likely to vaccinate their child than other respondents (22.2% and 10.9%, *P* = 0.035). Respondents who partially or fully agreed with the epidemiological guidelines were more likely to vaccinate their child than other respondents (30.7% and 12.5%, *P* < 0.001). Child age was also positively correlated with vaccination acceptance (*P* = 0.008); a higher percentage of respondents with a child in the 12-18 years group intended to vaccinate their child (27.1%) compared with the respondents with a child in the 5-11 years group (13.0%) or respondents with a child younger than five years (11.8%). Finally, respondents whose child had been vaccinated according to the national immunization program schedule were more likely to vaccinate their child against COVID-19 than respondents whose child had not been fully vaccinated (19.9% and 3.0%, *P* < 0.001). On the other hand, respondents’ sex, city of surveying, place of residence, household member number, education, and monthly household income were not significantly associated with vaccination intention. Other characteristics that were not associated with vaccination intention included previous COVID-19 infection by a household member or child, respondents’ post-vaccination side effects, and child comorbidities.

**Table 1:** Intention to vaccinate their child according to respondents' characteristics

			Vacination intention (%)			
	N (%) (unweighted)	Missing data N (%)	yes	hesitant	no	P*
All participants	872		18.50	46.30	35.20	
Survey center		0 (0.0)				0.410
Zagreb	446 (51.2)		21.12	44.38	34.04	
Split	231 (26.5)		14.36	51.74	33.90	
Osijek	195 (22.4)		14.43	43.43	42.14	
Sex		0 (0.0)				0.154
male	225 (25.8)		19.87	50.00	30.12	
female	647 (74.2)		17.02	42.32	40.66	
Age (years)		0 (0.0)				0.009
18-30	153 (17.6)		17.45	34.45	48.10	
31-45	616 (70.6)		14.53	49.36	36.11	
>45	103 (11.8)		31.24	44.37	24.40	
Place of residence		3 (0.3)				0.922
urban	595 (68.2)		18.84	45.66	35.50	
rural	274 (31.4)		18.21	47.97	33.82	
Household members		0 (0.0)				0.998
2–4	600 (68.8)		18.59	46.29	35.12	
>4	272 (31.2)		18.35	46.30	35.35	
Education level		0 (0.0)				0.837
secondary or lower	365 (41.9)		18.74	46.46	34.80	
tertiary or higher	507 (58.1)		17.27	45.50	37.23	
Monthly household income (euros)		127 (14.6)				0.595
<2000	436 (50.0)		21.25	47.15	31.61	
>2000	309 (35.4)		16.90	46.44	36.66	
Household member history of COVID-19		9 (1.0)				0.073
yes	546 (62.6)		15.65	45.24	39.11	
no	317 (36.4)		23.97	47.84	28.19	
Vaccinated against COVID-19		12 (1.4)				<0.001
yes	524 (60.1)		29.19	48.18	22.63	
no	336 (38.5)		3.17	45.18	51.32	
Post-vaccination side effects		350 (40.1)				0.747
yes	419 (48.1)		27.71	49.71	22.58	
no	103 (11.8)		33.97	45.31	20.73	
Post-vaccination side effect type		459 (52.6)				0.271
local	188 (21.6)		28.08	53.69	18.23	
systemic	44 (5.0)		25.08	33.68	41.24	
local and systemic	181 (20.8)		26.11	51.81	22.08	
Adherence to epidemiological measures		0 (0.0)				0.035
not completely	281 (32.2)		10.91	46.65	42.45	
completely	591 (67.8)		22.20	46.13	31.67	
Agreement to epidemiological measures		0 (0.0)				<0.001
not partially or completely	340 (39.0)		12.51	45.19	42.31	
partially or completely	532 (61.0)		30.72	48.57	20.70	
Child’s age (years)		0 (0.0)				0.008
<5	405 (46.5)		11.83	43.71	44.46	
5-11	286 (32.8)		13.03	50.95	36.02	
12-18	181 (20.8)		27.08	43.90	29.03	
Child’s comorbidities		45 (5.2)				0.527
yes	85 (9.8)		17.06	44.36	38.58	
no	742 (85.1)		20.00	48.39	31.61	
Child’s history of COVID-19		79 (9.1)				0.261
yes	207 (23.7)		23.91	54.36	21.73	
no	586 (67.2)		18.85	44.86	36.29	
Child regularly vaccinated		1 (0.1)				<0.001
yes	798 (91.5)		19.92	47.44	32.61	
no	73 (8.4)		3.02	34.15	62.84	

*χ^2^ test, Rao-Scott correction.

### Ordinal logistic regression analysis of childhood vaccination intention

Predictors of childhood vaccination acceptance were also identified with ordinal logistic regression. Predictors used in the model included respondents’ age, COVID-19 vaccination status, adherence to the epidemiological measures, agreement with the epidemiological measures, respondents’ child age, and child’s regular vaccination according to the national immunization program schedule (Table 2). Respondents who did not intend to vaccinate their child against COVID-19 were set as the reference group. Respondents who were vaccinated against COVID-19 were 4.1 times more likely to vaccinate their child against COVID-19 (95% CI 2.5-6.7, *P* < 0.001). Furthermore, respondents who partly or fully agreed with the epidemiological measures were 2.3 times more likely to vaccinate their child (95% CI 1.3-3.9, *P* = 0.003). Similarly, respondents with a child in the 12-18 years age group were 1.2 times more likely to vaccinate their child (95% CI 1.1-1.5, *P* = 0.038). Finally, respondents whose child had been regularly vaccinated were 3.8 times more likely to vaccinate their child (95% CI 1.7-5.9, *P* = 0.001). Respondents’ age and adherence to the epidemiological measures were not significantly associated with vaccination intention when taking into account the effects of other predictors in the model.

**Table 2:** Ordinal logistic regression model predicting childhood vaccination acceptance. Adjusted odds ratio corresponds to the number of times the participants were more likely to vaccinate their child when compared with the reference group. *P* value corresponds to the probability of obtaining the stated adjusted odds ratio value if no association between the respective predictor and child vaccination intention is present*

Predictor	aOR (95% CI)	p
Age (years)		
18-30 (ref)	1	
31-45	0.74 (0.32-1.69)	0.472
>45	1.29 (0.46-3.61)	0.626
Vaccinated against COVID-19		
yes	4.11 (2.51-6.72)	<0.001
no (ref)	1	
Adherence to epidemiological measures		
not completely (ref)	1	
completely	0.98 (0.56-1.72)	0.939
adequacy of epidemiological measures		
not partially or completely (ref)	1	
partially or completely	2.28 (1.33-3.90)	0.003
Child’s age (years)		
<5 (ref)	1	
5-11	1.09 (0.61-1.96)	0.775
12-18	1.23 (1.06-1.48)	0.038
Child regularly vaccinated		
yes	3.83 (1.74-5.86)	0.001
no (ref)	1	

*Abbreviations: aOR – adjusted odds ratio, CI – confidence interval, ref – reference group.

## Discussion

To our knowledge, this is the first study in Croatia that assessed parents’ attitudes on COVID-19 immunization in children. Similar to recently published studies from other countries (30,31), our respondents mostly had hesitant or negative attitudes toward childhood COVID-19 vaccination in Croatia. It is not easy to determine the main reason for the low vaccination acceptance rate, and many address the question as a complex issue. The most frequent explanations for vaccination hesitancy are the novelty of the vaccine, potential side effects, safety concerns, and the possibility of the vaccinated child developing severe COVID-19 despite vaccination (30-32).

Results of our bivariate analysis suggest that older respondents were more inclined to vaccinate their child against COVID-19. This finding is in line with the results of other studies (30,31,33). However, after adjusting for respondents’ vaccination status, we did not find a significant association between the child vaccination intention and age. Accordingly, our results also suggest that vaccinated parents were more likely to vaccinate their child against COVID-19. Interestingly, almost 23% of the vaccinated respondents did not intend to vaccinate their child, which could be a consequence of a lower risk for severe COVID-19 in children (6). Bivariate analysis also suggested a positive correlation between parents’ agreement with the epidemiological measures and the intention to vaccinate their child. This finding points toward a positive association between the respondents’ trust in the health care system and childhood COVID-19 vaccination acceptance. However, as the adherence to and agreement with the epidemiological measures were self-reported variables, this finding should be further explored. Additionally, as expected, respondents whose child had been vaccinated according to the national immunization program schedule were more inclined to vaccinate their child against COVID-19. Notably, the vaccine refusal rate in Croatia has recently increased (23). An important example is a lower rate of regular childhood immunization with the measles-mumps-rubella vaccine in Split and Osijek (34). This tendency, if it continues, could be one of the factors leading to an increasingly negative trend in the COVID-19 vaccination rate in children. We also found that parents with a child in the 12-18 age group were more likely to vaccinate their child. This result is interesting when considering a higher susceptibility to SARS-CoV-2 infection and a higher risk of severe COVID-19 presentation in younger children (3,4).

Interestingly, parents’ attitudes toward childhood vaccination were not significantly different in Zagreb, Split, and Osijek, which contradicts the lower adult vaccination rates recorded in Split (35). Similarly, we did not find a significant association between childhood vaccination acceptance rate and parents’ education, which contradicts the negative correlation between adult vaccination rates and education (36,37). We also found no significant association between parents’ intention to vaccinate their child and parents’ sex, place of residence, household size, or monthly income. These results agree with the results of various studies and demonstrate a lack of correlation between many of the sociodemographic parameters and acceptance rates of childhood COVID-19 vaccination (30,31,33). These findings also agree with the results of studies investigating the attitudes on vaccinating children in Croatia according to the national immunization program schedule (21,22). Parents’ and their child’s history of COVID-19 were also not associated with parents’ attitudes toward childhood vaccination, which could result from most respondents having had a mild form of COVID-19. Additionally, respondents’ post-vaccination side effects were not associated with their attitude toward childhood vaccination, which could be explained by relatively mild side effects in most parents. One of the most unanticipated findings of our survey is a lack of correlation between the parents’ attitudes toward childhood vaccination and children’s comorbidities. We found this result alarming due to the confirmed strong association between chronic disease and the likelihood of developing a severe COVID-19 (5). Interestingly, this result agrees with findings from similar studies and could be a consequence of parents’ lack of awareness of the severe COVID-19 risk in children with comorbidities (31,33). This potential lack of awareness should be explored in future studies focusing exclusively on children with chronic diseases.

Ordinal logistic regression analysis confirmed the already established relationship between parents’ attitude toward childhood vaccination and parents’ COVID-19 vaccination status, agreement with the epidemiological measures, children’s age, and children’s regular vaccination status. In this study, respondents’ adherence to the epidemiological measures was not a significant predictor of childhood vaccination acceptance due to its moderate positive correlation with agreement with the epidemiological measures, which was a stronger predictor of childhood vaccination acceptance. Similarly, parents’ age was not a significant predictor of a positive intention of childhood vaccination due to its moderate positive correlation with the COVID-19 vaccination status and child age. The strongest predictors for a positive attitude toward COVID-19 vaccination in children were parents’ COVID-19 vaccination status and children’s vaccination status according to the national immunization program schedule. All in all, the results of our analyses emphasize that future vaccination campaigns should primarily target unvaccinated parents, parents with younger children, and parents whose children suffer from chronic diseases.

We acknowledge several limitations of our study, the most important being the sampling design. Data collection in three largest regional centers in Croatia resulted in a good sampling coverage of the most populous counties in Croatia. Nevertheless, respondents from the counties gravitating toward other regional centers were likely underrepresented. Furthermore, data were collected in pediatric emergency departments at university hospitals, which also makes the study sample not nationally representative and introduces bias. However, these drawbacks were properly accounted for in the statistical analysis, which is why we believe they did not significantly alter the conclusions of this study. Another potential limitation of our study is recall bias. However, most of the survey questions potentially affected by recall bias examined recent events related to the COVID-19 pandemic. Thus, we believe that this did not notably impact the conclusions of our study. Future research should be based on a nationally representative sample that can be obtained, for example, through cooperation with primary pediatricians. In addition, as this study was conducted during the first few months of the COVID-19 vaccination campaign in Croatia, it would be beneficial to undertake another cross-sectional study in the future. This would allow for further insight into possible changes in parents’ attitude on childhood COVID-19 vaccination as a potential result of the COVID-19 vaccination campaign.
